# Kurzgefasstes Lehrbuch der Kinderheilkunde

**Published:** 1900-12

**Authors:** 


**Kurzgefasstes Lehrbuch der Kinderheilkunde.** Von Dr. CARL SEITZ. Pp. viii., 499. Berlin: S. Karger. 1901 [*sic*].

This is the second edition of a text-book on diseases of children, of which the first was published in 1893. It is a steady-going, humdrum, uninteresting book, with but little sense of perspective or proportion; the description of leucocythemia, for instance, occupies two pages, that of Asiatic cholera three pages, whilst that of adenoids is dismissed in a bare page and a quarter! The description of each particular disease is steadily worked through—anatomy, symptoms, diagnosis, prognosis, and treatment—in a jog-trot sort of way, so that whilst there is very little essential omitted, discussion of relationships of diseases, theories of their causation, the differences between adults and children in their liability to various diseases, the varying forms the same disease assumes in adults and children, the curious reactions of children to drugs—all that makes the study of children's diseases interesting—is disregarded. For instance, the fact that infantile scurvy often follows rickets is only mentioned. There is no general discussion of rheumatism in children, with its protean forms. Absence or deficiency of fat in food is not even mentioned as a factor in the etiology of rickets.

